# A prognostic index model to predict the clinical outcomes for advanced pancreatic cancer patients following palliative chemotherapy

**DOI:** 10.1007/s00432-015-1953-y

**Published:** 2015-03-20

**Authors:** Peng Xue, Lifei Zhu, Zhiyong Wan, Weiyi Huang, Ning Li, Donghui Chen, Jiong Hu, Haiyan Yang, Liwei Wang

**Affiliations:** 1 Nanjing Medical University, Nanjing, Jiangsu China; 2 Comprehensive Cancer Center, Shanghai General Hospital, Shanghai Jiaotong University, Haining Road 100, Shanghai, 200080 China

**Keywords:** Advanced pancreatic cancer, Prognostic index model, Palliative chemotherapy

## Abstract

**Purpose:**

To establish a prognostic index model for advanced pancreatic cancer patients receiving palliative chemotherapy based on clinical variables.

**Methods:**

The clinical data of 118 patients with advanced pancreatic cancer who received palliative chemotherapy between January 2006 and August 2013 in our center were retrospectively analyzed. Prognostic factors for overall survival were identified using Cox proportional hazards model. A prognostic index model was established by these pretreatment factors to predict prognosis. Kaplan–Meier estimation and log-rank test were performed to compare the overall survival difference between low-risk and high-risk group of patients.

**Results:**

Median overall survival time for all patients was 8.8 months [95 % confidence interval (CI) 7.0–10.6 months]. Multivariate analysis identified ECOG score = 2 (hazard ratio 2.03; 95 % CI 1.07–3.85; *P* = 0.030), CA19-9 levels of ≥1000 U/mL (hazard ratio 2.07; 95 % CI 1.09–3.92; *P* = 0.026), and CRP levels of ≥5 mg/L (hazard ratio 2.05; 95 % CI 1.06–3.96; *P* = 0.033) as independent poor prognostic factors for overall survival. For the three factors, ECOG score = 2, CA19-9 levels of ≥1000 U/mL, and CRP levels of ≥5 mg/L were allocated 1 point each. There were 84 (71.2 %) patients allocated to low-risk group with total score 0–1 point, and 34 (28.8 %) patients were categorized as high-risk group with total scores 2–3 points. The median overall survival for low-risk group and high-risk group was 9.9 months (95 % CI 6.8–13.0) and 5.3 months (95 % CI 4.1–6.5), respectively (hazard ratio 0.27; 95 % CI 0.14–0.52; *P* < 0.001). The estimated 1-year survival rates for low-risk group and high-risk group were 40.5 and 5.9 %, respectively (*P* < 0.05).

**Conclusions:**

A novel prognostic index model based on three clinical parameters was established to predict the prognosis of patients with advanced pancreatic cancer receiving palliative chemotherapy.

## Introduction

Pancreatic cancer is a devastating malignant disease represented by almost equal morbidity and mortality annually (Siegel et al. 2013). It is estimated to be the second leading cause of cancer-related death in USA around 2020 (Rahib et al. 2014). Although surgical resection is the only potential curative modality, only 10–20 % of patients were first diagnosed with resectable tumor (Heinemann et al. 2012). Even after curative resection, a large proportion of patients develop recurrence within 1 year (Katz et al. 2009; Barugola et al. 2009). The 5-year survival rate remains dismal (Stathis and Moore 2010).

In clinical practice, most patients present with locally advanced or metastatic disease at the first diagnosis (Heinemann et al. 2012; Stathis and Moore 2010). Palliative chemotherapy with gemcitabine (Burris et al. 1997; Heinemann et al. 2008) or S-1 (Okusaka et al. 2008; Ueno et al. 2013) based regimen for advanced pancreatic cancer (APC) has been established as the standard of care in recent years. However, the prognosis of patients receiving palliative chemotherapy varies depending on a number of clinical characteristics (Papadoniou et al. 2008). It is crucial to identify the subgroup of APC patients who would benefit from palliative chemotherapy (Philip et al. 2009). Previous studies have identified several prognostic factors for survival, such as pretreatment CA19-9 (Saad et al. 2002; Reni et al. 2009), C-reactive protein (CRP) (Haas et al. 2010; Wang et al. 2012), serum albumin levels (Maréchal et al. 2007), neutrophil-to lymphocyte ratio (NLR) (Xue et al. 2014), and performance status (Tas et al. 2013). However, the predictive value of these factors for palliative chemotherapy of APC patients is still controversial. Therefore, the development of an alternative method to predict the prognosis for APC patients is attracting more attention. Glasgow Prognostic Score (GPS) (McMillan 2013), a notable prognostic scoring method, which was developed a decade ago, represented a sensitive measure of the systemic inflammatory response and nutritional status of patient. Glen et al. (2006) verified its prognostic value in patients with inoperable pancreatic cancer. However, the host-related factor of GPS alone can hardly reflect the overall status of patients with pancreatic cancer. Therefore, we sought to develop a comprehensive and feasible prognostic index model to predict the prognosis for APC patients in daily practice.

In this study, we investigated multiple pretreatment variables that are easily accessible in clinical practice to predict the outcomes of APC patients receiving palliative chemotherapy. Furthermore, a prognostic index model derived from the independent prognostic factors was established to effectively identify the high-risk group of APC patients undergoing gemcitabine- or S-1-based palliative chemotherapy. It could be valuable for clinicians in decision making of treatment strategies and assessment of APC patients who may likely benefit from palliative chemotherapy.

## Materials and methods

### Patients and treatment

Clinical data of 145 consecutive APC patients, who received gemcitabine- or S-1-based palliative chemotherapy in our cancer center between January 2006 and August 2013, were retrospectively analyzed. Twenty-seven cases were excluded from this study due to deficient pathological or clinical data, and 118 patients who met the following inclusion criteria were included in our study: (1) patients with pathologically confirmed invasive ductal carcinoma of the pancreas, either by surgical resection or needle biopsy; (2) patients presented with locally advanced unresectable or metastasis disease diagnosed by computed tomography (CT) or magnetic resonance imaging (MRI); and (3) patients with available clinical data at the first administration of gemcitabine- and/or S-1-based palliative chemotherapy. All patients enrolled had signed informed consent previously for the purpose of clinical research. This study was approved by the Ethics Committee of Shanghai General Hospital, Shanghai Jiaotong University.

Palliative chemotherapy consisted of gemcitabine- or S-1-based regimens, including gemcitabine monotherapy (*n* = 68) (Burris et al. 1997), gemcitabine and cisplatin combination therapy (*n* = 8) (Heinemann et al. 2006), gemcitabine and oxaliplatin combination therapy (*n* = 12) (Louvet et al. 2005), gemcitabine and nab-paclitexal combination therapy (*n* = 4) (Von Hoff et al. 2013), gemcitabine and erlotinib combination therapy (*n* = 4) (Moore et al. 2007), and S-1 monotherapy (*n* = 22) (Okusaka et al. 2008; Ueno et al. 2013). The specific dosage and schedule of each regimen were adjusted by the physicians based on the individual patient’s general condition.

### Prognostic factors

The integrated clinical data included patients’ demographics, the medical treatment records, pathological reports, tumor-node-metastasis stage, imaging scan of body, and pretreatment laboratory data were collected for analysis. Fourteen clinical variables were chosen as potential prognostic factors, among which continuous parameters were divided into two categories according to the previous studies (Papadoniou et al. 2008; Haas et al. 2010; Xue et al. 2014; Tanaka et al. 2008) for the convenience of prognostic analysis as follows: age (<65 or ≥65 years), gender (male or female), Eastern Cooperative Oncology Group (ECOG) score (0–1 or 2), primary tumor location (head or body/tail), prior tumor resection (no or yes), distant metastasis (no or yes), levels of carbohydrate antigen 19-9 (CA19-9 <1000 or ≥1000 U/mL), carcinoembryonic antigen (CEA <5 or ≥5 ng/mL), C-reactive protein (CRP <5 or ≥5 mg/L), hemoglobin (<100 or ≥100 g/L), neutrophil-to-lymphocyte ratio (NLR <5 or ≥5), platelet to lymphocyte ratio (PLR <150 or ≥150), and albumin (<35 or ≥35 g/L).

### Statistical analysis

All of the analyses were performed using SPSS statistical software (version 17.0, SPSS Inc, Chicago, IL, USA). The primary end point of the study was overall survival (OS). OS was calculated from the initiation of palliative chemotherapy to the date of death for any reason or the last follow-up visit of patient. OS were estimated by the Kaplan–Meier method, and the difference in OS was compared by log-rank tests. Prognostic variables associated with OS were identified through univariate analysis by Cox regression models. The hazard ratio (HR) and 95 % confidence interval (CI) were calculated using Cox regression models. A two-tailed *P* value of <0.05 was considered statistically significant. The independent prognostic variables associated with OS were confirmed by multivariate analysis using Cox proportional hazards model. A prognostic index model was established based on independent variables that were significantly associated with OS in the multivariate analysis.

## Results

### Patient characteristics

A total of 118 consecutive patients with APC treated with first-line palliative chemotherapy between January 2006 and August 2013 were investigated. The median age of these patients was 62 years (range 34–82). Seventy-four patients (62.7 %) had relatively good general conditions with ECOG score 0–1. Forty-six patients (39.0 %) had pancreatic head carcinoma, while seventy-two patients (61.0 %) had carcinoma in the body and tail of pancreas. Twenty-four patients (20.3 %) had received primary pancreatic lesion resection before recurrence. Forty-six patients (39.0 %) had unresectable locally advanced lesion, while seventy-two patients (61.0 %) had distant metastatic disease. Of these patients, sixty-four (54.2 %) had liver metastasis, sixty (50.8 %) had celiac lymph node metastasis, and thirty-four (28.8 %) had ascites or peritoneum metastasis. Twenty-two patients (18.6 %) received S-1 monotherapy; other ninety-six patients (81.4 %) received gemcitabine-containing regimen treatment, among which sixty-eight had received gemcitabine monotherapy and twenty-eight had received gemcitabine-based combination therapy. Patients’ baseline characteristics are shown in Table 1.

**Table 1:** Baseline characteristics of 118 patients with advanced pancreatic cancer (APC)

Characteristic	Median (range)	Number (%)
Age (years)	62 (34–82)	
Gender		
Male		86 (72.9)
Female		32 (27.1)
PS score		
0–1		74 (62.7)
2		44 (37.3)
Primary tumor location		
Head		46 (39)
Body and tail		72 (61)
Recurrent or unresectable disease		
Unresectable		74 (79.7)
Recurrent		24 (20.3)
Metastasis		
Liver		64 (54.2)
Lymph node		60 (50.8)
Peritoneum		34 (28.8)
Palliative first-line regimen		
Gemcitabine containing		96 (81.4)
S-1 containing		22 (18.6)
NLR		
<5		84 (71.2)
≥5		34 (28.8)
PLR		
<150		76 (64.4)
≥150		42 (35.6)
CA19-9 (U/mL)	601.8 (0.6–2084)	
CEA (ng/mL)	34.9 (0.4–770)	
CRP (mg/L)	14 (0.2–135)	
ALP (IU/L)	163.7 (36–707)	
Hemoglobin (g/L)	111 (63–156)	
Albumin (g/L)	39.7 (28–48.7)	
TB (mg/dL)	15.8 (6–97.9)	
AST (IU/L)	32.1 (9–116)	
ALT (IU/L)	25.9 (5–101.7)	

### Univariate and multivariate analysis of prognostic factors

Univariate analysis of potential prognostic factors associated with OS in this cohort showed that ECOG score of 2, unresectable disease, distant metastasis, CA19-9 levels of ≥1000 U/mL, CRP levels of ≥5 mg/L, and NLR ≥5 were significantly associated with poor OS (*P* < 0.05). Performing the subsequent multivariate analysis, a total of three factors, including ECOG score = 2, CA19-9 levels of ≥1000 U/mL, and CRP levels of ≥5 mg/L, were identified as independent prognostic factors for poor OS in APC patients following palliative chemotherapy (Table 2).

**Table 2:** Univariate and multivariate analyses of prognostic factors in APC patients

Variable	No. of patients (%)	Median OS (months)	Univariate analysis			Multivariate analysis		
			Hazard ratio	95 % CI	*P* value	Hazard ratio	95 % CI	*P* value
Age (years)								
≥65	40 (33.9)	8.8 (5.179–12.421)	1	0.632–2.253	0.585			
<65	78 (66.1)	8.5 (5.808–11.192)	1.194					
Gender								
Female	32 (27.1)	6.3 (4.144–8.456)	1	0.673–2.777	0.388			
Male	86 (72.9)	8.9 (6.796–11.004)	1.367					
ECOG score								
2	44 (37.3)	5.5 (4.351–6.649)	1	0.255–0.863	0.015	1	0.260–0.933	0.030
0–1	74 (62.7)	9.9 (6.880–12.920)	0.470			0.493		
Primary tumor location								
Body and tail	72 (61)	7.3 (5.142–9.458)	1	0.632–2.170	0.617			
Head	46 (39)	8.9 (3.578–14.222)	1.171					
Recurrent or unresectable disease								
Unresectable	74 (79.7)	6.9 (5.288–8.512)	1	0.165–0.848	0.018	1	0.193–1.107	0.083
Recurrent	24 (20.3)	13.8 (8.862–18.738)	0.374			0.463		
Distant metastasis								
Yes	72 (61)	6.9 (5.430–8.370)	1	0.275–0.972	0.041	1	0.475–1.857	0.856
No	46 (39)	12.7 (9.336–16.064)	0.517			0.939		
CA19-9 (U/mL)								
≥1000	38 (32.2)	5.3 (4.020–6.580)	1	0.194–0.662	0.001	1	0.255–0.918	0.026
<1000	80 (67.8)	9.9 (6.689–13.111)	0.359			0.484		
CEA (ng/mL)								
≥5	78 (66.1)	8.8 (6.965–10.635)	1	0.443–1.583	0.585			
<5	40(33.9)	7.2 (0–16.750)	0.837					
CRP (mg/L)								
≥5	54 (45.8)	5.9 (4.373–7.427)	1	0.268–0.890	0.019	1	0.253–0.945	0.033
<5	64 (54.2)	10.6 (6.576–14.624)	0.488			0.489		
Hemoglobin (g/L)								
<100	18 (15.3)	4.5 (3.331–5.669)	1	0.228–1.064	0.072			
≥100	100 (84.7)	8.9 (6.664–11.136)	0.493					
NLR								
≥5	34 (28.8)	5 (3.118–6.882)	1	0.215–0.786	0.007	1	0.272–1.029	0.061
<5	84 (71.2)	9 (4.343–13.657)	0.411			0.529		
PLR								
≥150	42 (35.6)	9.7 (8.155–11.245)	1	0.524–1.792	0.919			
<150	76 (64.4)	7.2 (5.086–9.314)	0.969					
Albumin (g/L)								
<35	24 (20.3)	6 (2.945–9.055)	1	0.314–1.308	0.222			
≥35	94 (79.7)	9 (5.496–12.504)	0.641					
Regimen								
Gemcitabine containing	96 (81.4)	8.5 (6.657–10.343)	1	0.740–3.260	0.245			
S-1 containing	22 (18.6)	8.9 (1.469–16.331)	1.553					

### Overall survival

**Fig. 1:**
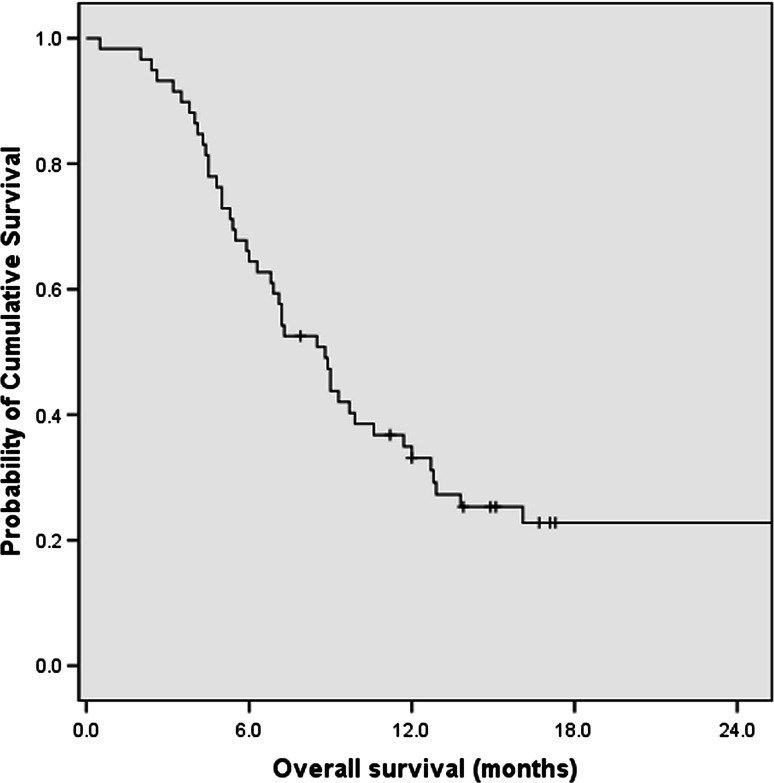
Overall survival (OS) curve for all the APC patients

With median follow-up period of 8.5 months (range 0.5–65.8), 88 patients (74.6 %) were reported dead at time of last follow-up. The median OS time for all the 118 patients was 8.8 months [95 % confidence interval (CI), 7.0–10.6 months] since initiation of palliative chemotherapy (Fig. 1). The 1- and 2-year survival rates were 32.2 and 10.2 %, respectively.

**Fig. 2:**
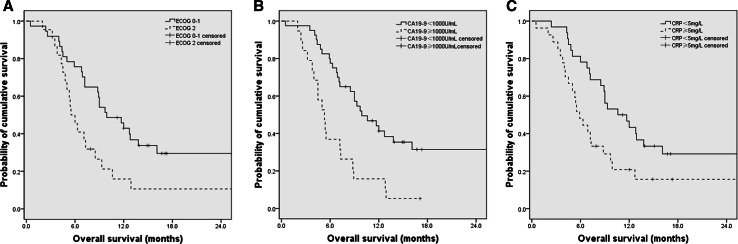
Overall survival of patients according to ECOG scored (**a**), CA19-9 value (**b**), and CRP value (**c**)

To compare OS of patients with different profiles of prognostic factors, we divided patients in subgroups according to the independent prognostic factors identified in the multivariate analysis, including ECOG score, CA19-9 levels, and CRP levels, and compared the Kaplan–Meier curves for OS by log-rank test (Fig. 2). The median OS was 5.5 months (95 % CI 4.4–6.6) in ECOG score 2 group and 9.9 months (95 % CI 6.9–12.9) in ECOG score 0–1 group [hazard ratio (HR) 0.47; 95 % CI 0.26–0.86; *P* = 0.015]. For patients with CA19-9 levels of ≥1000 versus <1000 U/mL, median OS was 5.3 months (95 % CI 4.0–6.6) versus 9.9 months (95 % CI 6.7–13.1) (HR 0.36; 95 % CI 0.19–0.66; *P* = 0.001). The median OS for patients with CRP levels ≥5 mg/L was 5.9 months (95 % CI 4.4–7.4) compared with 10.6 months (95 % CI 6.6–14.6) in patients with CRP levels <5 mg/L (HR 0.49; 95 % CI 0.27–0.89; *P* = 0.019).

### Prognostic index model

**Fig. 3:**
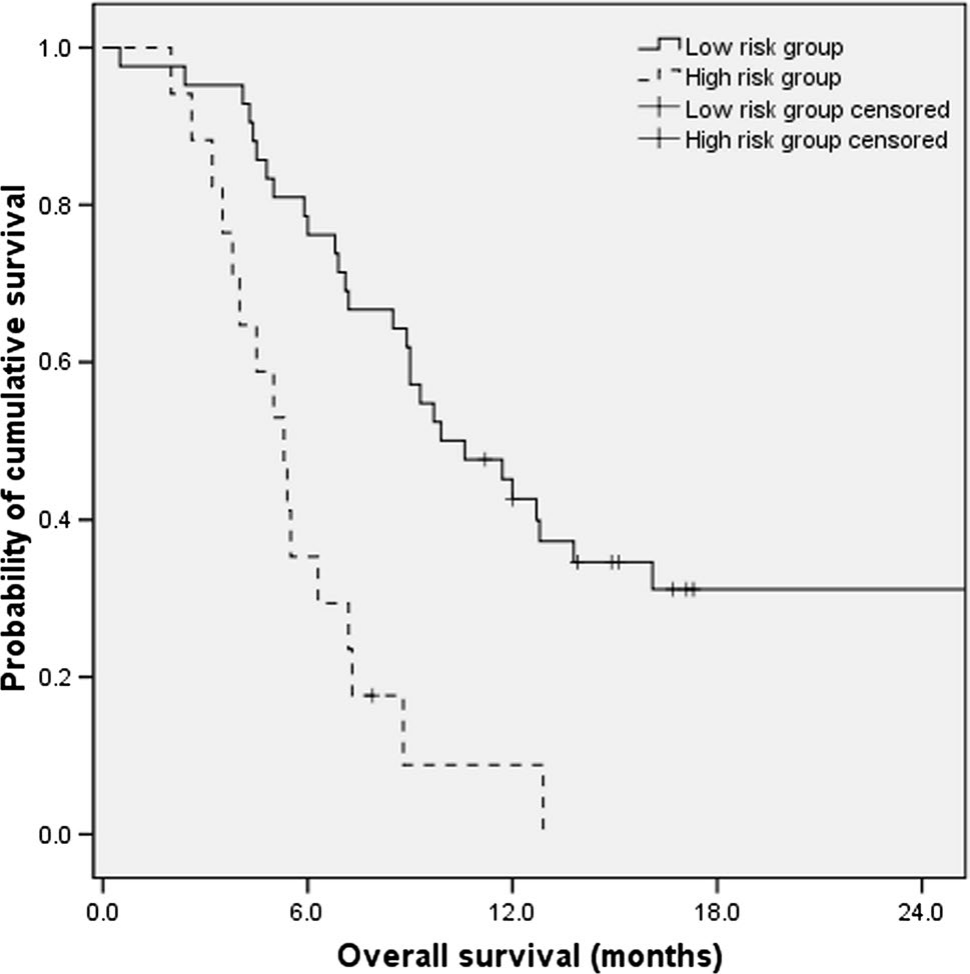
Overall survival of patients by prognostic index risk group

The pretreatment ECOG score, CA19-9 levels, and CRP levels identified as independent prognostic factor by multivariate analysis were used to establish the prognostic index model. Table 3 shows the criteria of prognostic index model as follows: ECOG score 2, CA19-9 levels of ≥1000 U/mL, and CRP levels ≥5 mg/L were allocated 1 point each; ECOG score 0–1, CA19-9 levels of <1000 U/mL, and CRP levels <5 mg/L were allocated 0 point each. The total score ranging from 0 to 3 was categorized into two prognostic index risk groups as defined as follows: low-risk group, 0 or 1 point; high-risk group, 2 or 3 point. There were 84 (71.2 %) patients allocated to low-risk group, and 34 (28.8 %) patients were categorized as high-risk group. The median OS for the low-risk group was 9.9 months (95 % CI 6.8–13.0), which was significantly longer than that of 5.3 months (95 % CI 4.1–6.5) in high-risk group (HR 0.27; 95 % CI 0.14–0.52; *P* < 0.001). The estimated 1-year survival rates for low-risk group and high-risk group were 40.5 and 5.9 %, respectively (*P* < 0.05) (Fig. 3).

**Table 3:** Criteria of the prognostic index model

Risk factors	Points
ECOG score	
2	1
0–1	0
CA19-9 levels (U/mL)	
≥1000	1
<1000	0
CRP levels (mg/L)	
≥5	1
<5	0

## Discussion

The incidence of pancreatic cancer has gradually increased in developing countries in recent years (Ma et al. 2013). Several clinical trials confirmed that gemcitabine- or S-1-containing palliative chemotherapy possess similar efficacy and safety for patients with APC; nevertheless, the prognosis of APC patients remains dismal (Stathis and Moore 2010; Burris et al. 1997; Heinemann et al. 2008; Okusaka et al. 2008; Ueno et al. 2013; Michl and Gress 2013). It is clinically relevant to identify the prognostic factors for APC patients in determination of the treatment strategy.

Previous studies have reported several prognostic factors related to poor outcomes for APC patients, including elevated pretreatment levels of CA19-9 (Saad et al. 2002; Reni et al. 2009), CRP (Haas et al. 2010; Wang et al. 2012; Pine et al. 2009), and LDH (Haas et al. 2010; Tas et al. 2001), increased ratio of neutrophil to lymphocyte (Xue et al. 2014), poor performance status (Tas et al. 2013), and status of initial unresectable disease (Xue et al. 2014). However, the prognostic factors reported were different in various studies. In the current study, we devised a convenient prognostic index model by retrospectively analyzing various clinicopathological factors and pretreatment parameters in APC patients receiving palliative chemotherapy. Three factors, including ECOG score of 2, CA19-9 levels of ≥1000 U/mL, and CRP levels ≥5 mg/L, were identified as independent adverse prognostic factors for OS in APC patients in our cohort. Creating a prognostic index model with these three factors, patients classified as low risk with this model showed significant survival benefit from gemcitabine- or S-1-based chemotherapy, with a median survival duration of 9.9 months and 1-year survival rate of 40.5 %, which was much better than the patients categorized as high risk, with median survival duration of 5.3 months and a 1-year survival rate of 5.9 %. Based on these values, palliative chemotherapy of gemcitabine- or S-1-containing regimen showed no significant survival benefit for the patients in high-risk group. As to the low-risk group of patients, it should be noted that those who have fewer poor prognostic factors may do well even without chemotherapy. Nevertheless, this prognostic index model would help oncologists to avoid excessive medical treatment for APC patients in high-risk group; meanwhile, it is urgent to develop novel therapeutical strategies for these intractable high-risk groups of APC patients.

 Previous studies have proposed various prognostic models for APC patients (details in Table 4). Hamada et al. (2014) recently designed a nomogram derived from analysis of their prospectively collected 531 patients with inoperable pancreatic cancer receiving palliative chemotherapy, which contained six potential prognostic factors of age, sex, ECOG PS score, tumor size, regional lymph node metastasis, and distant metastasis status. This nomogram provided improved ability to predict clinical outcome for each patient with APC. Yi et al. (2011) devised a risk-stratified prognostic model derived from 298 APC patients who received gemcitabine-based chemotherapy, which consists of four prognostic factors, including serum CRP levels, albumin levels, metastasis to liver, and ascites dissemination. The survival outcomes differed remarkably according to the prognostic model stratification. However, only 84.9 % patients in their study had definite pathological diagnosis of ductal adenocarcinoma. Maréchal et al. (2007) also reported a prognostic index consists of three variables of KPS, weight loss, and AST for APC patients who received gemcitabine-containing chemotherapy. Using this index model, patients were categorized into three groups with significantly different survival time. By contrast, three variables of ECOG score, CA19-9 levels, and CRP levels identified in our prognostic index model represent the general condition, tumor burden, and systemic inflammatory reaction of patient, respectively, making our result more close to clinical practice. Consistently, previous study has demonstrated that these three prognostic factors are significantly associated with the survival of a cohort of 103 consecutive patients with APC (Ueno et al. 2000). The median OS of this cohort was 3.2 months in contrast to 8.8 months in this study, and about 30 % of the patients had died within 2 months from the beginning of systemic chemotherapy. This may be because patients with locally advanced APC were excluded from this study. Additionally, the chemotherapeutic regimen used in this cohort varied among patients, having only a few patients treated with gemcitabine monotherapy which is not consistent to current clinical practice. Because of the disparity of categorization criteria for the continuous parameters, such as CA19-9 and CRP, between studies (Yi et al. 2011; Maréchal et al. 2007; Ueno et al. 2000), universally accepted cutoff values of these parameters have yet established. This underscores the importance of conducting further study to elucidate this issue. Our data suggests that the cutoff value defined in this study based on previous studies and our clinical practice could be utilized successfully to predict prognosis for APC patients. Obvious limitations should be addressed that the sample size of the current study is relatively small compared with other previous studies. Furthermore, the reliability of the predictive model developed in retrospectively single center should be externally validated using another independent cohort data.

**Table 4:** Published studies relating to the prognostic relevance by risk groups in APC patients

References	*n*	Treatment regimens	Variables (cutoff value)	Median overall survival time (by risk group)
Ueno et al. (2000)	103	Fluorouracil-based, gemcitabine, cDDP, docetaxel, epirubicin, irinotecan	CRP (5 mg/dL) CA19-9 (10,000 U/ml) ECOG PS(2)	5.2 months (good prognostic group) 2.6 months (intermediate prognostic group) 1.4 months (poor prognostic group)
Glen et al. (2006)	187	NA	CRP (10 mg/L) Albumin (35 g/L)	8.0 months (GPS 0 group) 4.3 months (GPS 1 group) 2.3 months (GPS 2 group)
Maréchal et al. (2007)	99	Gemcitabine based	KPS (90) Weight loss (10 %) AST (53 IU/ml)	356 days (A group) 212 days (B group) 80 days (C group)
Yi et al. (2011)	298	Gemcitabine based	CRP (1.2 mg/dL) Albumin (3.5 g/dL) liver metastasis Ascites dissemination	10.0 months (low-risk group) 6.7 months (intermediate-risk group) 4.4 months (high-risk group)
Hamada et al. (2014)	531	Gemcitabine based	Age, sex, ECOG PS, tumor size Lymph node metastasis Distant metastasis	17.5 months (very low-risk group) 13.7 months (low-risk group) 8.9 months (high-risk group) 5.5 months (very high-risk group)

*NA* no reported

In this study, all the APC patients have received either gemcitabine- or S-1-based chemotherapy. Although the chemotherapy regimens differed among patients in our study, it is unlikely that the treatment regimens would influence the prognosis of patients. Ueno et al. (2013) demonstrated in GEST study that efficacy of gemcitabine, S-1, or gemcitabine/S-1 combination therapy for patients with APC were not statistically different. In addition, previous meta-analysis studies (Heinemann et al. 2008) showed no significant improvement in OS for gemcitabine-based combinations over gemcitabine monotherapy. Furthermore, in our results of univariate analysis for different treatment regimens, the prognosis of patients received gemcitabine- or S-1-containing regimens showed no statistical difference (HR 1.55; 95 % CI 0.74–3.26; *P* = 0.245).

In conclusion, our study identified three independent adverse prognostic factors, ECOG, CA19-9, and CRP, in patients with APC who had received palliative chemotherapy. A prognostic index model calculated based on these factors was developed to stratify patients with low and high risk of poor prognosis. These readily accessible pretreatment parameters of patients and prognostic index model could assist clinicians to identify high-risk patients and propose individualized therapeutic approach to APC patients in clinical practice.

## Abbreviations

APC: Advanced pancreatic cancer
NLR: Neutrophil-to-lymphocyte ratio
PLR: Platelet-to-lymphocytes ratio
CA19-9: Carbohydrate antigen 19-9
CEA: Carcinoembryonic antigen
CRP: C-reactive protein
LDH: Lactate dehydrogenase
TB: Total bilirubin
AST: Aspartate transaminase
ALT: Alanine transaminase
ALP: Alkaline phosphatase
ECOG PS: Eastern Cooperative Oncology Group performance status
GPS: Glasgow Prognostic Score
CT: Computed tomography
MRI: Magnetic resonance imaging
OS: Overall survival
HR: Hazard ratio
CI: Confidence interval
